# Experimental data on behavioral, hepatosomatic, gonadosomatic indeces and total lipid of mud crab, Scylla olivacea at different velocity levels

**DOI:** 10.1016/j.dib.2019.104205

**Published:** 2019-07-02

**Authors:** Taufik Muhammad, Shahrul Ismail, Mhd Ikhwanuddin, Ambok Bolong Abol-Munafi

**Affiliations:** a Institute of Tropical Aquaculture, Universiti Malaysia Terengganu, 21030, Kuala Nerus, Malaysia; b School of Ocean Engineering, Universiti Malaysia Terengganu, 21030, Kuala Nerus, Malaysia; c STU-UMT Joint Shellfish Research Laboratory, Shantou University, Guangdong, China

**Keywords:** Aquaculture, Biochemical changes, Comparison between sexes, Hydrodynamic, Portunid crab

## Abstract

The data collected in the present work correspond to the behavioral, Hepatosomatic Index (HSI), Gonadosomatic Index (GSI) and total lipid analysis between male and female mud crabs, *Scylla olivacea* at different water velocities. A total of 56 immature male and female crabs were used in this data article. The important criteria for estimating the selective habitat facing by *S. olivacea* is a considerate of (1) the behavioral range in response to abiotic factors (and how it adapt ontogenetically) and (2) the movement of the crab under wild velocities situations. This work purposes to recognize the performance, locomotion rate and escaping capability of *S. olivacea* under stagnant and flowing water situations and to discuss the significance of horizontal walking to habitat choice. The collective outcomes clearly show that the locomotor activities and escaping capabilities of *S. olivacea* were influenced by water flow in the mangrove habitats. For the HSI data, velocities of 20 cm/s were the highest increased mean HSI percentage and highest mean HSI percentage in males and females was recorded on the end of the experiment. For GSI percentage of male and female crabs, 20 cm/s dominates the highest increases mean GSI, followed by 60, 40 and 0 cm/s. For total lipid percentage, the results showed that, the mean total lipid of hepatopancrease, muscle and gonad were increased at the beginning and decreased at the final in each water velocities except for 20 cm/s over a culture period of 60 days. Velocities of 20 cm/s were the highest increased mean total lipid percentage followed by 40, 60, and lastly 0 cm/s. The high flow velocities inhibit the production of hepatopancrease and gonad, in terms of nutrients from food used to endeavor the stress condition faced.

**Table:** Specifications table

Subject area	*Agriculture and Biological Sciences: Aquatic Sciences*
More specific subject area	*Physiology and Ecology: Eco-biological chemistry*
Type of data	*Table form*
How data was acquired	*Hatchery works and dissection of hepatopancreas, muscle and gonad tissues as well as biochemical analysis*
Data format	*Raw, filtered and analyzed*
Experimental factors	*Four different levels of water velocity*
Experimental features	*Investigation of 108 of immature male and female crabs by calculated male and female movement using Solomon coder software and counted time requirement for crab to flee and measured their HSI and GSI at novel simulator design between flow test and Re-circulating Marine Aquaculture System (RMAS)*
Data source location	*Institute of Tropical Aquaculture, Universiti Malaysia Terengganu*
Data accessibility	*With the article*

**Table:** 

**Value of the data** • Information regarding a cultured animal's velocities stress is vital to describe the velocities suitability of the animal. • To assess the effects of various water velocities levels during culture growth. Rapid fluctuation in such as water velocities can cause stress to the aquatic animals. • Data on the locomotor and escaping capabilities of crabs are critical to understand when and how much they impact by velocities tolerance. • In crustaceans, the hepatopancreas is an important organ for the absorption and storage of nutrients, and can synthesize digestive enzymes for food digestion [1]. It is necessary to assess the effects of various water velocities level during culture at growth-phase on hepatopancreas accumulation percentage in the bodies of crabs. • GSI is the ratio of crab gonad weight to total body weight and it is particularly helpful in identifying days and percentages of gonad maturation for reproduction [2], [3]. Data on GSI of crabs are critical to understand when and how much they are impacted by velocity tolerance and how they affect the overall functioning of gonad maturation. • Lipids play an important role during the development of decapod crustaceans, not only as a source of energy, but also as sources of essential nutrients [4]. The reason for slow growth during culture in hatchery may be lack of information on total lipid percentage at hepatopancreas which act as fuel tissue and regarding water velocities as natural habitats involved in tidal situations. • This data can be valuable for possible joint collaboration with the other institution as well as for comparison to other environmental conditions in portunid crabs [5].

## 1 Data

This dataset provides detailed information on: (Sheet 1) male and female locomotor activities (Tables 1 and 2) (Sheet 1) How much time require for crab to flee (Tables 3 and 4). (Sheet 2) Hepatosomatic Index (HSI) and Gonadosomatic Index (GSI) percentage (Tables 1–4) (Sheet 3) Total lipid percentage of hepatopancreas, muscle and gonad of mud crab *Scylla olivacea* (Tables 1–6).

## 2 Experimental design, materials, and methods

An experiment was performed with the PVC pipe prototype. Four different water velocities were tested by adjusting the valve of the prototype; following in Treatments 1, 2, 3, and 4 with flows of 0, 20, 40, and 60 cm/s, consistently. The experiment was run in Re-circulating Marine Aquaculture System (RMAS) with video camera fitted overhead the simulator system [6], [7] and flow meter was installed in the system. The water channel of the PVC pipe was fitted and the length was 114 cm long × 5 cm wide; for all the treatments, the pipe was filled with treated brackish water to a depth of 4 cm. Mimicking flow was produced in the pipe by an electric pump, with a capacity of 6000 L/h. Availability of dye movements in the pipe revealed the water current across the pipe. In a mangrove habitat, the mean range of the water velocities is about 10 cm/s [8], [9]. Therefore, four different water velocities such as 0, 20, 40, and 60 cm/s were applied as free stream velocities copying typical natural flow situations. A Marsh–McBirney Model 2000 Flo-Mate made in the US was the free-moving flow-meter applied in this experiment. Velocities calculated as (cm/s) were analyzed using the electromagnetic field, with a decimal of ±5 cm/s. For the present data, 48 crabs were alone located in the pipe for 24 h earlier to the experiment. Before experiment started, the water flow was fix to the preferred level, ambient illuminations were discarded, and the simulator was permitted to work for 15 min. The crabs were documented for 15 min as they walked in the center of the pipe. The flow quickness was manipulated to the following level, and the sequence described above was repeated. Flow quickness were increased from 0 cm/s to 60 cm/s. In this trial, the camera was installed above the pipe to give an accurate view of the pipe. Walking rate from the video records for at least six crabs in each trial for the 15-min period and the process was repeated until movements of 48 crabs had been recorded. The recorded video (MPEG-TS Video File) was analyzed using behavioral coding software Solomon Coder [10].

For the GSI, HSI and total lipid investigation, 56 crabs were individually placed in the pipe 24 h prior to the start of the experiment. Before the experiment started, the water flow was set to the exact level, normal light was not measured, and the pipe was allowed to run for 60 days. After 30 days, three crabs from each treatment were sampled for HSI, GSI and total lipid analysis. The experiment was performed at the hatchery and laboratories of the Institute of Tropical Aquaculture, Universiti Malaysia Terengganu (UMT). Immature male and female mud crabs were taken from a local crab hunter in Terengganu, Malaysia. The mud crabs were carried back to UMT alive in a basket. In the current data, the following equipment was used for culturing: 30 units of PVC pipe prototype, two holding tanks (3000 L), and one treated brackish water (20 ppt) stoking tank (3000 L). A work by Ikhwanuddin et al. [11] on the size of maturity (Carapace Width with 50% maturity size - CW_50_) for males at maturity was 8.97 cm CW for *S. olivacea*; given that, immature male crabs with a carapace width <8.97 cm were chosen in this work. Moreover, only immature female *S. olivacea* were selected. Previous methods by Ikhwanuddin et al. [12], the size at maturity (CW_50_) recorded for female *S. olivacea* was 9.06 cm. Hence, immature female crabs with an external carapace width <9.06 cm and a small, white-pale abdominal flap were selected in this work. The crabs’ body weight (BW) and carapace width (CW) were noted before the experiment begin. The size of the crabs was determined as the external CW, which was the place between the tips of the 9th anterolateral spines of the carapace. For each crab, the CW was determined with a Vernier caliper (decimal, 0.01 mm), while BW was determined by a digital balance (decimal 0.01 g). During the data experiment, the crabs were cultured in brackish water at 20 ppt. When measured, the water temperature was found to be around ±26 °C, but the temperature was not include in this experiment. Maintenance work done included siphoning the feces, uneaten food, sediment, and metabolic waste at the bottom [13], [14]. This was done in the morning before new fish (*Decepterus* sp.) was given. Fifty percent of the water was changed every two days. The gonads (ovaries and testes) and hepatopancreas of the crabs were subsequently weighed; remaining tissues were stored at −80 °C for later biochemical analysis of lipid according to the previous methods by Folch et al. [15]. In brief, the dry weight sample (hepatopancrease, muscle and gonad) were weight ± 1 g and noted as W1. The sampled then were put into thimble, weight and labeled as W2. Solvent 50 ml of petroleum ether were put into thimble and boiled 15 minute at temperature 90 °C. The thimbles were rinsed with petroleum ether for 30 minute. After that the thimble were kept dry for 10 minute. The total lipid residues accumulated at bottom of thimble were put inside the oven for 2 h at 100 °C for fully dried. The thimble then was weight as W3. Total lipid percentage was calculated by this formula:

$$\frac{\text{W}3-\text{W}2}{\text{W}1}\times 100$$

## 3 Statistical analyses

Changes between mean walking rates (irrespective of motion way) of male and female crabs were verified using one-way ANOVA, followed by a Tukey HSD test. Agreement with the expectations of data distribution normality and homogenized was evaluated using standard normal plots and Cochran's C test. The variances in the frequency distributions of walking frequency between rising and descendent flow test within a flow treatment were tested using Kolmogorov-Smirnov test. Data relating to walking frequency within a flow treatment were classified into *post hoc* categories of 20 cm/s intervals. The hepatopancreas, gonads and muscle were sampled from at least three crabs for the subsequent analyses. Differences between genders and mean HSI, GSI and total lipid percentage were analyzed by using One-way ANOVA and Tukey HSD Test through application of IBM SPSS Statistics Version 22 software and Microsoft Office Excel 2016.
